# Temperature Asymmetry Analysis between Left and Right Wrist with Sensory and Infrared Thermography

**DOI:** 10.3390/ijerph191610240

**Published:** 2022-08-18

**Authors:** Alejandra García Becerra, Jesús Everardo Olguín Tiznado, Jorge Luis García Alcaraz, Claudia Camargo Wilson, Juan Andrés López Barreras, Julio Cesar Cano Gutiérrez, Rosa Blanca Garcia-Rivera

**Affiliations:** 1 Department of Industrial Engineering, Tecnológico Nacional de México/IT Ciudad Guzmán, Ciudad Guzman 49100, Mexico; 2 Faculty of Engineering, Architecture and Design, Universidad Autónoma de Baja California, Ensenada 22860, Mexico; 3 Department Industrial Engineering and Manufacturing, Autonomous University of Ciudad Juarez, Ciudad Juárez 32310, Mexico; 4 Faculty of Chemical Sciences and Engineering, Autonomous University of Baja California, Tijuana 22390, Mexico; 5 Faculty of Administrative and Social Sciences, Universidad Autónoma de Baja California, Ensenada 22890, Mexico

**Keywords:** fingers, infrared thermography, physiological variables, repetitive work, temperature recovery

## Abstract

This article reports a thermal analysis of the wrists to analyze the behavior and recovery of skin temperature after 20 min when performing a highly repetitive movement, and two thermography methods (sensory and infrared) and research groups were compared. The tests were carried out with 44 participants who performed a repetitive task for 10 min and integrated into two groups, of which 22 were trained workers from a maquiladora company and were analyzed with sensory thermography, and the other 22 were in the laboratory with infrared thermography with undergraduate students. The study area is the left and right hand, specifically the wrists. The proposed hypothesis is that people with some musculoskeletal problems have a decrease in temperature when starting repetitive tasks and thermal asymmetries, which measurements were recorded at 0, 10, 15, and 20 min after the task was finished. Findings indicate that the temperatures in both wrists behave similarly. The workers reached higher temperatures, and the centigrade degrees of asymmetry difference were also higher. The variable with influence on the temperature was fractured in the arm. After thermally analyzing the temperature behavior between the wrists of both hands, it is concluded that there is an increase in temperature after finishing a repetitive task, and it does not stabilize after 20 min. Both thermography methods observed that the asymmetries are greater than 0.5 °C, detecting the possible pathology of carpal tunnel syndrome.

## 1. Introduction

This paper reports a temperature analysis in the wrist area by comparing two methods based on thermography. The need to evaluate two techniques arises to compare, through analysis, the reliability, cost, and benefit of the methods, to know if there are significant differences between results obtained, as well as to compare some of their characteristics and benefits.

In humans, the normal range for core temperature (CT) is 36.1 to 37.8 °C, and the skin is the organ responsible for maintaining and preserving it. Body temperature is controlled by the hypothalamus, which balances heat generation and loss, which is connected to the pituitary gland at the brain’s base, near the brainstem’s end.

The hypothalamus acts as a negative feedback loop, part of the autonomic nervous system. Specialized neurons in the hypothalamus act as thermoreceptors and constantly monitor the temperature of the blood against an internal set point [1]. Among the skin functions, the most important is the detection of sensation due to nerve endings, which react to temperature, touch, pressure, and vibration, as well as temperature regulation and evaporation control [2].

An important characteristic of the skin is the relative power of its surface to emit heat by radiation, meaning that it is a poor thermal reflector, but it can absorb and emit energy [3,4]. However, the human skin has its place among other radiators of its emission power, and the value depends on the technique to measure the skin’s temperature using radiometric instruments [5].

The increase in body temperature causes a greater amount of emitted radiation, and thus, an increase in vascularization, characteristic of many pathological changes such as inflammation or neoplasms with increased metabolic activity. This leads to an increase in temperature detected by an infrared thermal imaging camera [6,7].

Infrared thermography (IRT) is a functional diagnostic tool for some diseases and serves as a screening test for various conditions. IRT technology has its historical basis in developing digital underwater temperature recorders used to measure temperature in natural environments and with potential applications in areas such as oceanography, marine ecology, and industry, among others [8].

IRT applications in health areas have been widely reported: for example, for diagnosing the Raynaud’s Phenomenon (RP) [9,10,11], in which the temperature of the coldest finger is assessed and is useful as a complement; and for timely detection in a change of inflammatory activity [12]. Additionally, in diseases such as carpal tunnel syndrome, scanning the skin surface temperature in the relative area helps to make an earlier diagnosis [13,14].

IRT has also been used to analyze symptoms related to repetitive work, including pain, swelling, stiffness, numbness, tingling, clumsiness, loss of coordination and strength, skin discoloration, and temperature differences. The body parts involved are usually the extremities of the upper body, but there are also disorders of the neck, shoulder, elbow, forearm, wrist, and hand [15].

Previous reports of the application of IRT indicate that it served as an aid in diagnosing carpal tunnel syndrome (CTS) in 44 samples, differentiating healthy hands from those with pathologies [16]. A study was carried out on workers in a manufacturing company in which 75% of the people who performed a highly repetitive movement were diagnosed with some injury through IRT [17]. Temperature asymmetry is an aspect to consider in determining an abnormal pathology in chronic diseases [18,19]. Medical digital thermal imaging (DTI) is a passive, non-invasive, nonionizing, complimentary diagnostic, and real-time monitoring technique that enables visualizing and quantifying changes in skin surface temperature [20,21].

Another novel technique used is sensory thermography, which bases its operation on sensor monitoring to capture temperature, which can record temperature second by second, depending on where the sensors are placed [22]. These devices are based on a Negative Temperature Coefficient (NTC) or a Positive Temperature Coefficient (PTC) with a temperature range from 0 to 40 °C, accuracy ±0.3 °C, and measurement accuracy of 0.1 °C [5]. Thermistors are passive semiconductors sensitive to temperature; they exhibit a large change in electrical resistance when subjected to a slight change in temperature. NTCs thermistors decrease resistance when there is an increase in temperature and PTCs increase resistance when the temperature goes up [23].

Unlike infrared thermography, sensory thermography is a technique in an evolutionary process that bases its operation, as its name implies, on sensors placed on the skin, measuring its temperature second by second. The equipment to perform this technique costs approximately USD 1000, as opposed to a thermal imager that costs approximately USD 1500 with a resolution of 160 × 120. In addition, an advantage of sensory thermography is that during the protocol, the humidity of the place where the tests are carried out does not have to be controlled, and it does not necessarily have to be a laboratory or place with special conditions.

Considering that in both tests, the color of the skin does not significantly influence the results [24], the objective of this article is to carry out a comparative analysis of the two techniques to know their reliability and to know the feasibility of the application, given the difference in cost they have and the ease of access that could be had to them. Both techniques are used in the medical field to diagnose certain accumulated trauma disorder diseases, such as CTS, and are considered non-invasive. In addition, this study aims to test if there is a difference between methods in highly trained persons with and untrained persons. Through this study, it is expected that managers of health centers, hospitals, and companies have empirical and analytical evidence to decide what techniques to invest in to carry out this analysis, given that there is a difference in price for the cameras and tools involved.

### Study Justification

Thermography is used in medicine for different pathological diagnoses, such as oncology, pain, arthritis, rheumatism, surgeries [7], Raynaud phenomenon [4,25,26], vascular disorders (diabetes, deep vein thrombosis), and surgery [19]. However, a limitation is that the temperature’s reliability varies concerning the zone evaluated [27].

Different studies have focused on analyzing the wrists’ temperature, such as carpal tunnel syndrome diagnosis when performing repetitive activities [13,14,28]. Symptoms include pain, swelling, stiffness, numbness, tingling, clumsiness, loss of coordination and strength, skin discoloration, and differences in temperature [29,30,31,32,33,34,35]. Generally, the most affected parts are the upper limbs [36].

An aspect to consider in determining an abnormal pathology in chronic diseases is temperature asymmetry [20,36,37]. Differences in skin temperature of more than 0.5 °C between the sides of the body or extremities suggest underlying pathology [20], and even studies report evidence that technically healthy subjects showed temperature asymmetry of only 0.4 °C [37]. Thermal asymmetry in the areas of the body indicates a dysfunction that is due to the high correlation that exists between both sides of the body, while the increase in heat is mainly associated with inflammation or infection [38].

Thus, in this study, temperature increases indicate an abnormality, and even a difference in temperature between an affected and unaffected extremity equal to or greater than 1 °C is a risk factor [10], as occurs with patients studied with carpal tunnel syndrome [39]. Those high temperature gradients above 0.5 °C are related to dysfunction [40,41].

During repetitive work, the muscles increased their temperature, demonstrating a high local muscle activity proportional to the work time. Areas such as the dominant arm have shown a higher temperature of 2 °C to 4 °C, unlike the other areas [29], requiring special attention.

For example, one indicator of CTS is skin asymmetry; in a study, it is declared that in healthy subjects, the asymmetry between the extremities maintains a maximum value of 0.5 °C [37]; however, it assumes that for an anomaly to exist, the degree of asymmetry is 0.3 °C [19] and it also indicates that in people with abnormalities in one hand, the temperature difference between the two should be less than 1 °C [39]. Finally, in another study, the use of thermographic images was compared and indicated that for the temperature difference between a healthy shoulder and a shoulder with tendinitis, the asymmetry temperature is greater than 0.5 °C [42].

## 2. Materials and Methods

### 2.1. Stage One: Sampling

For the analysis of both techniques, 44 participants were considered. The sensory thermography sample was carried out with operators in a manufacturing company located in Hermosillo (Mexico), where highly repetitive tasks are performed in winding cables. On the other hand, the analysis with infrared thermography was carried out with students and workers in a Higher Education Institution (HEI) laboratory by voluntary participation. The sensory tested group were 15 females with age 27.1 ± 3.4 and 7 males with age 28.8 ± 6.5 years old; while the infrared group were 15 females with age 24.7 ± 6.6 and 7 males with 21.4 ± 1.6, looking to have similar groups. Please see Table 1 for a full sample description.

The task was the process of winding cables by the operators of the manufacturing company. Finally, to carry out tests on both groups, it was necessary to explain the study’s objective and the importance of their participation and ask them for their collaboration and commitment to obtain data reliability.

### 2.2. Stage Two: Obtaining the Information

In the sensory thermography analysis, the tests lasted approximately 3 weeks. In the development of the tests, the operators were selected who work operating the wiring assembly process, which has highly repetitive movements, and some of the operators have injuries in the wrist area. In addition, two important variables to study were defined, the temperature and the number of cycles, understanding the number of cycles as the number of pieces the operator can make each time.

The relationship with the simulated tests were analyzed with infrared thermography, and volunteer workers and students from an HEI were recruited. The experiment lasted approximately 3 months and was carried out in September–November 2019 in a laboratory of the IES. The participants were registered in a database as they performed the tests; a time of 10 min performing the repetitive movement and the recovery time for obtaining the thermograms were defined.

### 2.3. Stage Three: Protocol

**Figure 1:**
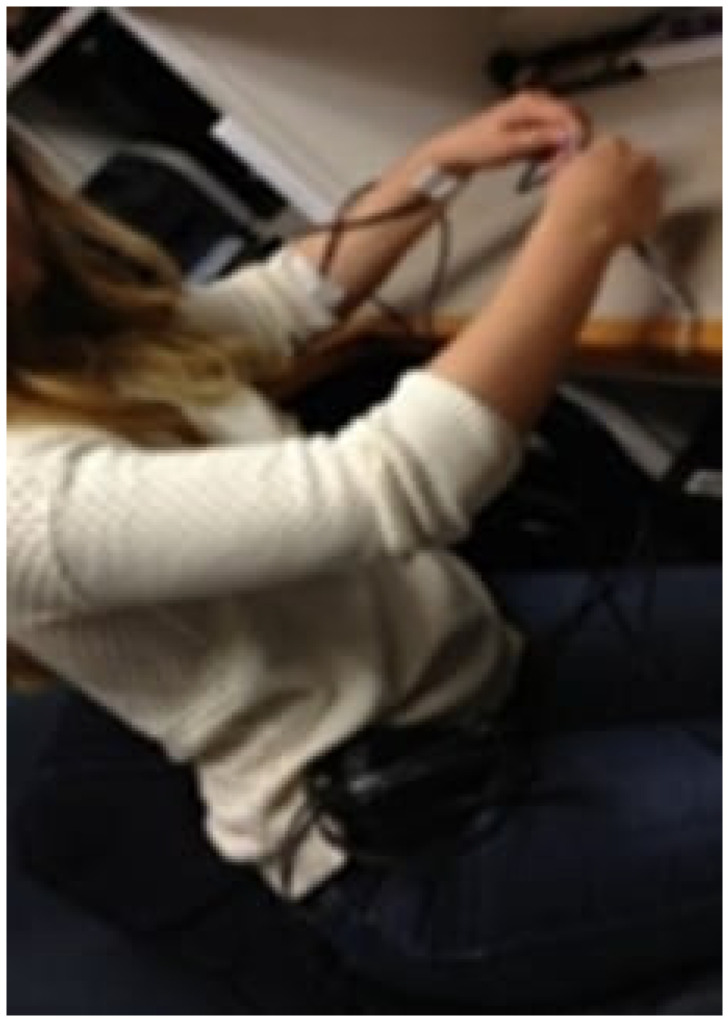
Sensory thermography, the procedure to carry out the tests with sensory thermography, placed the sensors in the wrist and elbow.

For sensory thermography, the sensors are placed on the skin of the operators, in the wrist and elbow, as shown in Figure 1, recording the operators’ temperature second by second. The materials and equipment used were as follows:

- A personal computer with Intel Core i5 (3rd Gen) 3340 M at 2.7 GHz with 4 Gb of DDR3 SDRAM memory.
- A sensor at 1600 MHz; two calibrated Sköll sensory thermographs (Ensenada, BC, Mexico), based on an NTC thermistor, whose resistance is highly dependent on temperature, with a range of 0 to 40 °C, precision ± 0.3 °C, resolution of 0.1 °C to collect the data [5].
- For programming the sensory thermographs, the Akela program is used (Longmont, CO, USA).

For each method, the groups integrate 7 men and 15 women, and before the test, demographic information was obtained from the person(s) regarding their age, body weight, blood pressure problems, trauma problems such as fractures, habits such as smoking or drinking, and accumulated time of exposure to the development of the operation, among others. Additionally, persons were asked to refrain from doing any type of exercise for 20 min before [43,44] and they were asked to refrain from drinking alcohol or smoking before the test since smoking produces a decrease in temperature [44].

The protocol for the sensory thermography tests was as follows:

1. Program the Sköll thermographs using the Akela program.
2. Once the pertinent information was collected, the ambient temperature in the study laboratory was taken to maintain it in an ideal range of 20–25 °C [43,44,45]. Although it is a wide temperature range, according to information from the company’s human resources department, these are the real conditions where operators perform operations on the production lines.
3. After the stabilization time, the operator was asked to sit in a chair, and the Sköll thermographs were immediately placed on both hands, specifically on the wrist area (in the region of the median nerve). Once the Sköll thermographs were placed, the operator was asked to rest his arms on a flat table adjusted to the height of the ribs for a space of 20 min and with a pelvic angle of 90° [44]. This is done at the beginning and the end of the test.
4. Place the Sköll thermographs on the wrist area (on the left and right hand).
5. Emulation of assembling the cuff of a t-shirt is a highly repetitive operation carried out in the company for one hour.
6. Surveillance and identification during the test about anomalies or pain presented by the operators.
7. Withdrawal of the Sköll thermographers and concentration of the data obtained in Microsoft^®^ Office Excel for manipulation and interpretation.

For infrared thermography, the equipment and materials used in this test were as follows:

- An infrared thermography camera Thermal brand FLIR^®^ (Wilsonville, OR, USA) E25 (Longwave IR camera at 7.5 to 13 µm spectral range, Focal Plane Array sensor of 160 × 120, Noise Equivalent Temperature Difference of <100 mK at 30 °C and measurement uncertainty of ± 2% of overall reading) was used. The camera was calibrated by the FLIR company according to a standard calibration to manufacturer specifications and with 17,025 accreditations, because they are the only ones accredited to do that.
- The images were analyzed with the FLIR^®^ ThermaCAM Researcher Pro software v.2.10 from Teledyne FLIR company at Wilsonville OR (USA), which allows the drawing of regions of interest (ROI) and extracting the average temperatures from them. The ROIs used were the inner canthi of the eyes (for estimating body core temperature [20]), hands, and fingers.

**Figure 2:**
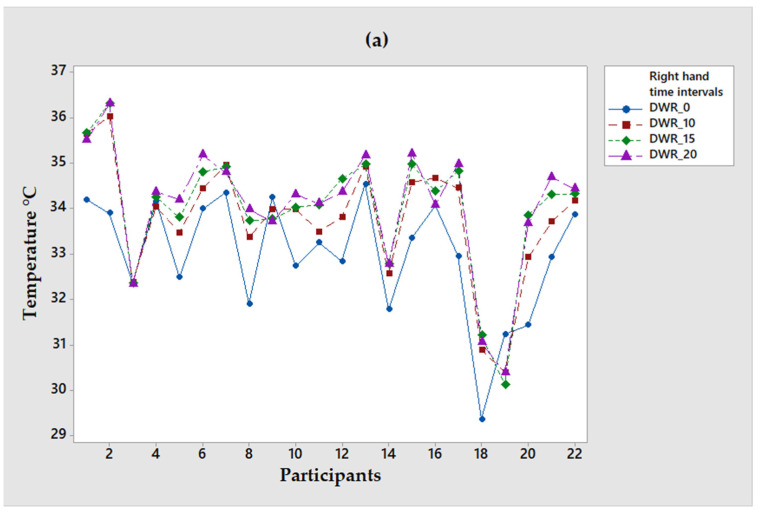

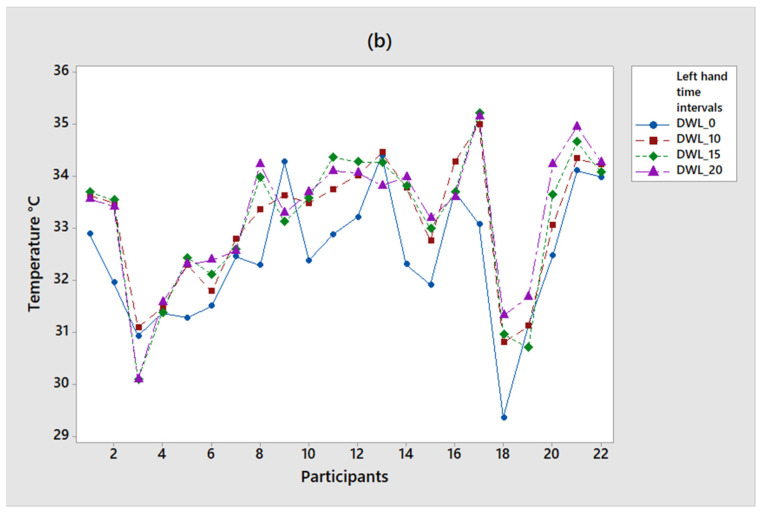
Temperature sample of participants with TS: (**a**) Wrist of the right hand and (**b**) Wrist of the left hand.

The procedure followed to obtain the information in this test was as follows:

- People were chosen to participate voluntarily in the emulated tests. A vital characteristic to consider was that they would be participants of productive age and all participants were registered in a database to keep track of them.
- In the laboratory where the tests were carried out, the temperature was controlled between 20 to 25 °C, and the relative humidity did not exceed 50% [42,46,47,48], replicating real factory conditions. For the IRT tests, the emissivity of the chamber was kept at 98%, which is the value for human skin [49,50].
- Each participant would be assigned approximately one hour to take the thermographic images. Thermograms were taken and recorded in a database.
- They explained the movement they had to carry out in which they emulated the movements of the operators of the manufacturing company for 10 min winding cables, which involved the 10 fingers of the hand. A thermogram was taken before starting the task, and the hands maintained the same position on a base (see Figure 2) after 10 min at the end of the repetitive activity. The hands were then kept at rest, and temperatures were recorded at 15 and 20 min.

### 2.4. Stage Four: Information Analysis

In this study, both techniques are compared, each with a group of equal participants. The first group is a manufacturing company’s workers performing highly repetitive movements in their operation. These tests were carried out with sensory thermography. The second groups were workers and students of an HEI, where the analysis was through emulated tests replicating the movements of the first group (workers), and it was through infrared thermography.

For the first group, temperatures were obtained with sensory thermography, where the temperature is recorded second by second. During this process, the workers kept the sensors attached to their skin while they performed their operation. The database and information were analyzed in Excel, where the temperature corresponding to minutes 0, 10, 15, and 20 were subsequently extracted.

For the second group, the tests were emulated, and the thermograms were obtained using the infrared thermography technique. The shots were made at 0, 10, 15, and 20 min. The images were analyzed using the Thermal Cam software to obtain temperatures, segmenting the image into different regions. The information collected was entered into an Excel database for analysis.

The results of both techniques are contrasted with the two groups of 15 participants of the female gender and 7 of the male gender; in total, there were 44 participants (22 in each group). To choose the participants, it was considered that they were approximately in the same range of the variables: age, gender, and BMI for these descriptive statistics were obtained. For each group, the difference in asymmetry between the areas of the wrists was obtained, and the normality tests and hypothesis tests were obtained.

To compare the two samples and thermography techniques, a normality test is performed on the data obtained; however, not all the variables follow a normal distribution, so non-parametric tests are used, such as the Kruskal–Wallis test, which allows one to identify whether the two data groups come from the same population or differ from each other. The calculations are made with a significance level of (*p* < 0.05) in the SPSS^®^ v.25 software.

The first analysis that is carried out is the descriptive statistics of the variables: age, body mass index, and the number of fractures that the participants had in the upper extremities. This is done in groups; the first group was for sensory thermography and the second group was for infrared thermography.

The second analysis is performed on the variables: age, body mass index, gender, and fractures against the temperature obtained at 0, 10, 15 and 20 min for both groups. These tests are also estimated with the Kruskal–Wallis test to analyze which variables influence the temperature of the wrist.

Subsequently, the average of these temperatures TS and TIR are separated by group. The fourth analysis was about the asymmetries that they presented between the right hand and the other hand, and this analysis was carried out separately for each of the groups to analyze which ones exceeded 0.5 °C since, according to Pichot [41], this is a sign of pathologies and to analyze in what period the greatest asymmetries were present.

To finalize the analysis, 5 random people from each group were considered, and their temperatures were graphed for each hand in each period (0, 10, 15, and 20 min) to analyze which minute maximum temperatures were reached.

## 3. Results

### 3.1. Sample Descriptives

Table 1 summarizes the information on age, body mass index (BMI), and the number of reported fractures for the 44 participants (15 female and 7 male for each method). In this case, it is observed that the infrared thermography group had fewer fractures on average than the sensory thermography group; however, this may be because the average ages in the infrared thermography group are much younger.

In this case, men have an average age for ST of 28.85 ± 6.5 years, and women, 27.13 ± 3.4 years; this variable was not statistically significant concerning temperature (*p* < 0.05). However, a relevant piece of information is that the company’s operators showed pain symptoms.

### 3.2. Statistical Analysis of Both Groups with the Two Techniques

When performing the statistical analysis to know the influence of temperature, it was found that this is not statistically significant (*p* < 0.05). However, a relevant fact is that the company operators indicated that they had pain symptoms. Likewise, it was found that there is no statistical evidence to determine the influence of the fracture variable on wrist temperature when highly repetitive activities are performed. However, a statistical relationship was found between the slow recovery of temperature with the gender of the people and with the BMI. The values of this test are observed in Table 2, where W represents the wrist of the hand, R and L represent the right position and the left position, while the values 0, 10, 15, and 20 are the minutes in which the temperature measurements are made, and α represents the *p*-value. Thus, for example, WR_10 refers to the temperature in the right wrist at minute 10.

All the participants in both groups were right-handed, and Table 3 illustrates the average maximum temperature for the right and left wrist at 0, 10, 15, and 20 min after starting the activity, where the two groups have been unified. In this case, it is observed that the right hand maintains an average temperature slightly higher than the left hand, without a statistically significant difference between them.

Table 4 indicates the average temperatures and standard deviation for each of the techniques analyzed in the two hands for the different periods in which the measurements are made. When comparing the left and right hands in each technique, it is observed that the temperature in the right and left hands were always higher using the sensory thermography technique. The maximum temperature was at 20 min with ST with a value of 34.1 °C ± 1.3 in the right hand. The IRT sample’s maximum temperature was 28.3 ± 1.5 at 15 min in the left hand. Here, it is concluded that the temperature is higher among the company’s workers by an average of 5.8 °C compared to the students and workers of (HEI) who carried out emulated tests.

The analyzed values of thermal asymmetry for the sensory thermography sample for the different periods are illustrated in Table 5, where it is observed that half of the sample exceeds 0.5 °C of asymmetry at the beginning of the experiment (that is, without performing repetitive movements). After 20 min, in most of the participants who already showed an asymmetry, the temperature increased by more than 0.5 °C.

Table 6 shows the comparison of asymmetry of the dominant and right hand using infrared thermography. The greatest difference in absolute value is found at minute 20; in addition, the highest values were observed in participants 4 and 17.

Figure 2 graphically illustrates the behavior of the temperatures of the ST group (of the 22 participants), and it is observed that the maximum temperature for the right wrist is 36.3 °C 15 min after starting the test. On the other hand, the maximum temperature recorded on the left wrist was 33.5 °C, which was recorded 15 min after the start of the test. In these graphs, the temperature remains stable in almost all groups after 10 min.

**Figure 3:**
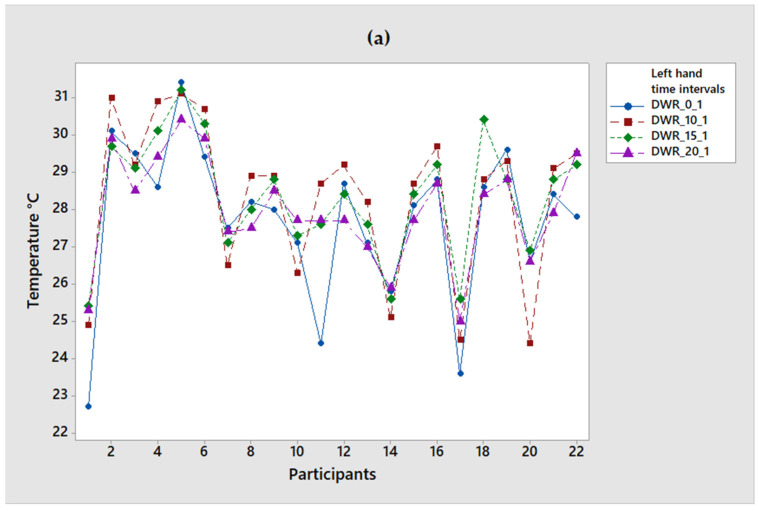

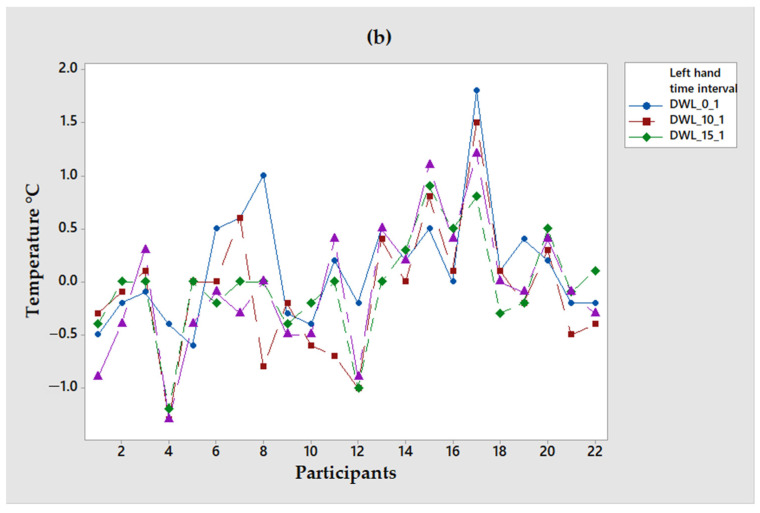
Temperature sample with IRT: (**a**) right-hand wrist temperature (**b**) left-hand temperature.

For the IRT the maximum temperature recorded was at 15 min with a value of 31.2 °C for both the right and left wrist, as illustrated in Figure 3. In these graphs, it can be seen that the temperature behavior remains irregular between each period, unlike the first group of workers.

### 3.3. Qualitative Comparison of the Two Techniques

After carrying out the qualitative analysis of the information of the ST and IRT techniques, Table 7 has been prepared, in which a comparison of said analysis is summarized. It is observed that the difference in the cost of the required equipment is approximately 500 dollars; in both tests, it is required not to consume alcohol, not smoke, nor play sports before applying it. However, the IRT requires controlled environments for its execution, and there is no physical contact with the participants.

Based on this comparison, administrators of hospital centers and medical managers in companies can better decide which technique and equipment to use to monitor workers’ health and diagnose anomalies that may occur early.

## 4. Discussion and Implications

One of the findings of this research shows that both techniques are useful for assessing skin temperature and identifying rapid changes in skin temperature, because they can predict the presence of musculoskeletal disorders [11,51]. Previous studies have shown that acute injury causes an increase in permanent blood flow, which implies increased metabolic activity, as demonstrated in this study [52].

This work has two objectives; the first is to compare the two thermography methods, and the second is to determine if there is a difference between a group of people who have been trained and have experience performing the activity (line operators) and another group who do not (university students). However, we agree that this research has a limitation, and in future research, both methods will be analyzed in the same working groups.

The results of the ST analysis (in company workers) show a temperature increase of up to 1.2 °C after 15 min of initiating repetitive motion with the right hand, which happens to be twice that of the left hand, which represented only a 0.6 °C temperature increase.

IRT has the ability to show temperature changes over a period of time, unlike sensory thermography, which measures the body’s behavior second by second. It is based on the heat emission of bodies above absolute 0, and it is an active tool for analyzing the behavior of temperature in the skin. [12]. With this technique, temperature gradients can be analyzed, but when performed by taking thermograms, these can be reflected in time lapses and not continuously, as in the case of sensory thermography. Therefore, it is more accurate and economical to use ST for this type of study.

During the process of obtaining data in a maquiladora company, acquiring the thermograms, that is, the humidity levels (45% to 60%), depended greatly on the climatic conditions. As well as the taking of thermal images, there was a restricted schedule in order not to affect the daily production goals for the company.

## 5. Conclusions

In this study, the behavior of the temperatures of the wrists in both hands when performing a repetitive activity was thermally analyzed through the analysis of the TS and IRT, and it was statistically verified that there is an increase in temperature after carrying it out and this does not recover after 20 min. In both thermography methods, asymmetries greater than 0.5 °C were observed; in neither case did the temperature decrease after repetitive activity.

In future work, it is proposed to analyze how long it takes for the skin temperature to fully recover for healthy people and people with some ATDs in the upper extremities. Likewise, it is proposed to analyze how many degrees of asymmetric difference exist between healthy subjects and subjects with some pathology. However, if in the study it is desired to know the temperature analyzed second by second, then sensory thermography is recommended, in addition to being more practical for evaluating operators within a company, since a deep protocol is not required. However, infrared thermography has advantages since it provides images of areas with higher temperatures, which can obtain the same data when analyzed.

## Figures and Tables

**Table 1:** Sample characterization.

Group	Gender	*N*	Age	BMI	Fractures
Sensorial	F	15	27.1 + 3.4	25.4 + 5.5	2
	M	7	28.8 + 6.5	28 + 4.8	10
Infrared	F	15	24.7 + 6.6	24.8 + 4.8	0
	M	7	21.4 + 1.6	25.6 + 4.5	1

**Table 2:** Kruskal–Wallis test.

	Stretch Techniques		Gender		BMI		Fractures	
	*p*	Decision	*p*	Conclusion	*p*	Decision	*p*	Conclusion
WR_0	0	Influential	0.241	Not influential	0.12	Not influential	0.004	Influential
WL_0	0	Influential	0.98	Not influential	0.07	Not influential	0.003	Influential
WR_10	0	Influential	0.212	Not influential	0.198	Not influential	0.006	Influential
WL_10	0	Influential	0.96	Not influential	0.066	Not influential	0.001	Influential
WR_15	0	Influential	0.371	Not influential	0.185	Not influential	0.007	Influential
WL_15	0	Influential	0.374	Not influential	0.103	Not influential Not influential	0.001	Influential
WR_20	0	Influential	0.29	Not influential	0.149	Not influential	0.01	Influential
WL_20	0	Influential	0.724	Not influential	0.09	Not influential	0.03	Influential

**Table 3:** The average temperature on the wrists of the total sample.

Time	Average Temperature °C	
	Right	Left
0	30.4 ± 3	30.1 ± 3
10	31 ± 3.2	30.7 ± 2.9
15	31.2 ± 3.2	30.7 ± 2.8
20	31 ± 3.4	30.6 ± 3

**Table 4:** Average temperature for each sample.

Time (min)	ST		IRT	
	Right	Left	Right	Left
0	33 ± 1.2	32.4 ± 1.2	27.8 ± 1.9	27.7 ± 2
10	33.8 ± 1.3	33.1 ± 1.18	28.2 ± 1.8	28.3 ± 2
15	34 ± 1.3	33.1 ± 1.3	28.3 ± 1.4	28.3 ± 1.5
20	34.1 ± 1.3	33.2 ± 1.2	27.9 ± 1.3	27.9 ± 1.4

**Table 5:** Asymmetry analysis for sensory thermography.

Period	Participants																					
	1	2	3	4	5	6	7	8	9	10	11	12	13	14	15	16	17	18	19	20	21	22
0	1.3	2	1.4	2.8	1.2	2.5	1.9	−0.4	0	0.4	0.4	−0.4	0.1	−0.5	1.5	0.4	−0.1	0	0.1	−1	−1.2	−0.1
10	2	2.6	1.3	2.6	1.2	2.7	2.2	0	0.4	0.5	−0.3	−0.2	0.5	−1.2	1.8	0.4	−0.5	0.1	−0.7	−0.1	−0.6	0
15	2	2.8	2.3	2.9	1.4	2.7	2.3	−0.2	0.6	0.4	−0.3	0.4	0.7	−1	2	0.7	−0.4	0.2	−0.6	0.2	−0.3	0.3
20	2	2.9	2.3	2.8	1.9	2.8	2.2	−0.2	0.4	0.6	0	0.3	1.4	−1.2	2	0.5	−0.2	−0.3	−1.3	−0.6	−0.3	0.2

**Table 6:** Asymmetry analysis for infrared thermography.

Period	Participants																					
	1	2	3	4	5	6	7	8	9	10	11	12	13	14	15	16	17	18	19	20	21	22
0	−0.5	−0.2	−0.1	−0.4	−0.6	0.5	0.6	1	−0.3	−0.4	0.2	−0.2	0.5	0.2	0.5	0	1.8	0.1	0.4	0.2	−0.2	−0.2
10	−0.3	−0.1	0.1	−1.3	0	0	0.6	−0.8	−0.2	−0.6	−0.7	−1	0.4	0	0.8	0.1	1.5	0.1	−0.2	0.3	−0.5	−0.4
15	−0.4	0	0	−1.2	0	−0.2	0	0	−0.4	−0.2	0	−1	0	0.3	0.9	0.5	0.8	−0.3	−0.2	0.5	−0.1	0.1
20	−0.9	−0.4	0.3	−1.3	−0.4	−0.1	−0.3	0	−0.5	−0.5	0.4	−0.9	0.5	0.2	1.1	0.4	1.2	0	−0.1	0.4	−0.1	−0.3

**Table 7:** Qualitative analysis between ST and IRT.

	Cost	Relevant Points	Invisibility Level
Sensory Thermography	USD 1000	Do not consume alcohol; No Smoking; Do not play sports before; There is no limitation in maintaining fixed humidity.	The sensors are affixed to the skin during the evaluation.
Infrared thermography	USD 1500 or more	Do not consume alcohol; Do not smoke; Do not play sports before; Maintain a specific humidity and luminosity in the place. The emissivity of the camera for the skin must always be 98%.	Contact with the participant is not necessary.

## Data Availability

Raw data are available under request from the corresponding and first authors.
